# A combination of phospholipids and long chain polyunsaturated fatty acids supports neurodevelopmental outcomes in infants: a randomized, double-blind, controlled clinical trial

**DOI:** 10.3389/fnut.2024.1358651

**Published:** 2024-06-13

**Authors:** Qiqi Ren, Xiaoyu Zhu, Jiancun Pan, Kaifeng Li, Yalin Zhou, Ying Lyu, Qinggang Xie, Yajun Xu

**Affiliations:** ^1^Heilongjiang Feihe Dairy Co., Ltd., Beijing, China; ^2^PKUHSC-China Feihe Joint Research Institute of Nutrition and Healthy Lifespan Development, Beijing, China; ^3^Department of Nutrition and Food Hygiene, School of Public Health, Peking University, Beijing, China; ^4^Beijing Key Laboratory of Toxicological Research and Risk Assessment for Food Safety, Peking University, Beijing, China

**Keywords:** brain development, anthropometry, early life nutrition, breastfeeding, bioactive substance

## Abstract

Phospholipids (PLs) and long-chain polyunsaturated fatty acids (LCPUFAs) are naturally present in breast milk and play important roles in promoting the growth of the infant. Several studies have investigated the effects of the combination of PLs and LCPUFAs on neurodevelopment. However, data on the effectiveness of infant formula containing both PLs and LCPUFAs on the neurodevelopment of infants is still scarce. This randomized, double-blind, controlled clinical study was designed to evaluate the effect of an infant formula enriched with PLs and LCPUFAs on growth parameters and neurodevelopmental outcomes in term infants up to 365 days of age. Infants were enrolled within 30 days of birth who were then randomly assigned to either a control group (*n* = 150) or an investigational group (*n* = 150). Both groups consist of cow’s milk-based formula which were generally identical in terms of composition, except that the investigational formula was additionally supplemented with PLs and LCPUFAs. The infants were followed for the first year of life. Breastfed infants were the reference (*n* = 150). Bayley Scales of Infant Development [3rd edition (Bayley-III)], Carey Toddler Temperament Scales (TTS), MacArthur-Bates Communicative Development Inventories (CDI), Single Object Attention and Free Play Tasks were used to evaluate neurodevelopmental outcomes of infant at 365 days of age. In addition, Ages and Stages Questionnaires (ASQ) were also conducted at 120, 180, and 275 days of age. Compared to breastfeeding, both infant formulas were well-tolerated and provided adequate growth, with no adverse events being reported throughout the study. Infants of the investigational group showed higher mean scores in Bayley-III cognitive performance (104.3 vs. 99.0, *p* < 0.05), language (106.9 vs. 104.5, *p* < 0.05), and motor skills (109.2 vs. 103.9, *p* < 0.05) compared the control group. Similar results were being reported for other developmental scales including TTS and ASQ. Notably, the test scores of infants fed the investigational formula were similar to those who were breastfed. Our results indicate that PL and LCPUFA supplementation may be beneficial for neurodevelopment of infants throughout the first year of life. Further studies are needed to investigation long-term effects PL and LCPUFA on neurodevelopment in early life.

## 1 Introduction

Breast milk contains a wide range of components that are considered the optimal source of nutrients for feeding infants (1). It provides numerous advantages such as cognitive development, defense against pathogens, digestion and absorption of nutrients, and a lower risk of chronic diseases in later life (2). However, various circumstances can hinder breastfeeding, resulting in a significant number of infants worldwide receiving partial or exclusive formula feeding (3). Hence, it is crucial to develop an ideal infant formula that closely matches the composition, functional properties, and health-based outcomes of breast milk in order to meet the healthy growth needs of infants. In recent years, there has been growing recognition of previously overlooked functional components in breast milk, such as complex lipids, bioactive proteins, and oligosaccharides, which have been incorporated into infant formulas (4). Additionally, there is increasing focus on studying the potential biological functional effects of these components when added to infant formulas.

Approximately 60% of the energy consumed in an infant’s first year of life is used for brain development, most of which is primarily allocated to the development of synthetic neuronal membranes and myelin. Lipids found in breast milk play a crucial role in providing the necessary energy and essential nutrients for the growth of the infant’s brain and nerves (5). Approximately 98–99% of lipids in breast milk are glycerides, while phospholipids (PLs) account for around 0.2–2.0% of the total lipids (6). PLs have greater bioactivity compared to the main energy-supplying glycerides and offer various benefits in neurodevelopment, gut health, inflammation, and cardiovascular disease (7). PLs can be extracted from sources such as milk, soybeans, egg yolk or marine organisms (8). Approximately 60–65% of milk PLs are attached to milk fat globule membrane (MFGM). The MFGM not only contains PLs, but also a large number of bioactive compounds such as sphingolipids, glycolipids, and glycosylated proteins (9). Breast milk is also a source of long chain polyunsaturated fatty acids (LCPUFAs), specifically docosahexaenoic acid (DHA) and arachidonic acid (ARA). These fatty acids were found that are the most abundant in the structure of nervous tissue (10). The provision of LCPUFAs through breast milk has a positive effect on the development of retinal and brain cortical function in infants (11).

Dietary PLs phospholipids and gangliosides have been found to enhance spatial learning and impact brain growth and composition in neonatal piglets (12). Furthermore, early clinical studies have confirmed the safety of bovine MFGM (bMFGM) or its components in infant formula. An increasing number of clinical trials have associated the inclusion of dietary MFGM with beneficial effects on neurodevelopment (13–15) and child behavior (16). There have also been several early clinical trials investigating the beneficial effects of LCPUFAs -fortified formula on children’s cognition (17, 18) and behavior (19). In addition, several clinical trials have investigated the effects of supplementing formula with bMFGM and LCPUFAs on infant neurodevelopment (14, 15, 20). However, data on the effectiveness of infant formula containing both PLs and LCPUFAs on infant neurodevelopment remain scarce and need to be supplemented in areas such as a longer feeding period up to 1 year of age for infants, a larger sample size, a more comprehensive neurodevelopmental assessment scale, and a more extensive control group design.

Consequently, further research is needed to fully understand and confirm the efficacy, mechanism of action, and long-term benefits of adding PLs from different sources, and LCPUFAs to infant formulas. The objective of the present study was to evaluate the neurodevelopmental outcomes at 12 months of age in healthy term infants who received formula supplemented with PLs and LCPUFAs as compared with infants receiving a routine cow’s milk-based formula.

## 2 Methods

### 2.1 Design and participants

Eligible infants were enrolled in this single-center, randomized, double blind, controlled, parallel-designed, prospective study from August 2020 to June 2022 in Jinhua, Zhejiang Province, China. This research was conducted in accordance with the Declaration of Helsinki, and approved by the Institutional Review Board of Shanghai Nutrition Society. This trial was registered at clinicaltrials.gov as NCT04508257. Informed consent was obtained from all subjects involved in the study. Acceptable participants were either exclusively formula-fed infants or breastfed infants. Participant inclusion criteria for participants were as follows: infants had to be at least 30 days old at the time of randomization, exclusively formula-fed for at least 3 days before randomization, born as singletons, with a gestational age between 37 and 42 weeks (36 weeks and 6 days was considered 36 weeks gestational age), a birth weight between 2,500 g to 4,000 g, and informed consent obtained from the parent or guardian for the infant’s participation in the study. Additionally, the parent or guardian agreed not to enroll the infant in any other interventional clinical research study while participating in this study. The exclusion criteria included individuals with a history of underlying metabolic or chronic disease, congenital malformation, or any other condition that, in the investigator’s opinion, could interfere with the infant’s ability to ingest food, normal growth and development, or the evaluation of the infant. Additionally, infants with evidence of feeding difficulties or formula intolerance, such as vomiting or poor intake, at the time of randomization were excluded (at the discretion of the investigator). Infants with a weight at visit 1 less than 95% of their birth weight [(weight at Visit 1 ÷ birth weight) × 100 < 95%] were also excluded. Furthermore, infants who were immunocompromised (according to a doctor’s diagnosis of immunodeficiency) or had known head/brain disease/injury, such as microcephaly or macrocephaly, were excluded.

The study period involved feeding and cognitive testing up to 365 days of age (Supplementary Table 1). The study consisted of 6 visits at specific time points: 30 (±7 days; enrollment), 90 (±7), 120 (±7), 180 (±7), 275 (±7), and 365 (±7) days of age. Participants in the formula feeding groups were given stage 1 formula until 180 days of age and then transitioned to stage 2 formula until 365 days of age. Cognitive tests were conducted by trained professionals at the study sites. All participant data was recorded in an electronic case report form (eCRF) by designated and trained study personnel. The study adhered to good clinical practices and was registered with ClinicalTrials.gov (NCT04508257).

### 2.2 Randomization and study group allocation

Eligible infants who were exclusively consuming their mother’s breast milk with the intent to continue through at least 4 months of age and continue with supplement food without any marketed infant formula were registered in the breast milk reference group. Infants whose mothers had chosen to exclusively feed them infant formula until 365 days of age were randomly allocated to either a control formula (*CF*) group or an investigational formula (IF) group with a ratio of 1:1 stratified by gender. The composition of two formulas were identical except that the IF was fortified with whey protein-lipid concentrate (Stage 1&stage 2: source of bMFGM; Lacprodan MFGM-10, Arla Foods Ingredients P/S), soybean phospholipids (Stage 1& stage 2: source of PLs; soybean phospholipids ingredient, Cargill), fungal-derived single cell oil (Stage 1& stage 2: source of ARA), and algal-derived single cell oil (Stage 1& stage 2: source of DHA; Table 1). Both formulas were produced in the same factory in China Feihe, using identical production and processing equipment. In addition to the added PLs and LCPUFAs, both formulas also contain a certain baseline level of these components. This is because the main ingredients of both formulas are raw cow’s milk, and PLs and LCPUFAs naturally exist in raw cow’s milk. The randomization scheme was generated using Version 9.4 of SAS^®^ statistical software. The identities of the specific products are blinded to participants, parents or legal guardians, support staff and investigators. Investigator will receive one sealed and numbered envelope for each participant containing the identification of the study product administration. The unblinding will occur to the statistical analyses team only after completion of statistical analyses. Upon database lock the treatment codes were transmitted to the statistical group generating the final analysis for incorporation into the study analysis datasets.

**Table 1:** Nutritional composition per 100 mL.

	Study formula (target values)			
	Stage 1		Stage 2	
	Control	Investigational	Control	Investigational
Energy, kcal	67	64	71	69
Protein, g ^1^	1.40	1.35	2.18	2.26
Casein, g	0.52	0.50	1.09	1.13
Whey, g	0.88	0.85	1.09	1.13
Carbohydrate, g ^2^	7.0	6.7	8.1	7.8
Lactose, g	6.8	6.5	7.8	7.5
GOS, g	0.45	0.45	0.64	0.68
Fat, g ^3^	3.3	3.4	3.1	3.1
Linoleic acid, g	0.53	0.53	0.37	0.37
α-Linolenic acid, mg	53	53	37	37
DHA, mg	0.1	12.2	0.1	15.0
ARA, mg	1.3	20.8	2.0	25.0
PLs, mg	20	67	19	70
PC, mg	6	22	6	24
PE, mg	5	21	5	23
PI, mg	2	6	2	6
PS, mg	2	6	2	6
SM, mg	5	12	4	11

GOS, galactooligosaccharide; DHA, docosahexaenoic acid; ARA, Arachidonic acid; PLs, Phospholipids; PC, Phosphatidylcholine; PE, Phosphatidylethanolamine; PI, Phosphatidylinositol; PS, Phosphatidylserine; SM, Sphingomyelin.^1^Sources of protein for control: skim milk and whey protein concentrate (WPC); and for investigational: skim milk, WPC, and whey protein-lipid concentrate (source of bMFGM protein; Lacprodan® MFGM-10, Arla Foods Ingredients P/S, Denmark).^2^Sources of carbohydrate for control and for investigational: lactose and GOS.^3^Sources of fat for control: base blend of sunflower, coconut, and flaxseed oils; and for investigational: base blend of sunflower, coconut, flaxseed oils, fungal-derived single cell oil (source of ARA), algal-derived single cell oil (source of DHA), whey protein-lipid concentrate (source of bMFGM phospholipids; Lacprodan® MFGM-10, Arla Foods Ingredients P/S, Denmark) and soybean phospholipids (source of phospholipids; soybean phospholipids ingredient, Cargill).

### 2.3 Study outcome measures

The Bayley Scales of Infant and Toddler Development, Third Edition (Bayley-III) evaluates infants and children from 1 to 42 months of age. The cognitive, language (receptive and expressive communication), and motor (fine and gross motor) domains were assessed by a trained evaluator. The social–emotional and adaptive behavior scales were assessed by parent questionnaire (21). The Bayley-III has been previously translated into Chinese (Mandarin), adapted for the Chinese population. The study aimed to assess infant neurodevelopment at 365 days of age using the Bayley -III as the primary outcome. In addition to the Bayley-III, the following instruments used were previously translated, revised, and adapted for use in Chinese populations: TTS, CDI, Single Object Attention and Free Play Tasks, and ASQ. The study analyzed the output of the TTS on 9 domains of infant temperament, including activity level, regularity, approach/withdrawal, adaptability, intensity, mood, persistence, distractibility, and sensor threshold. The Chinese (Putonghua) Communicative Developmental Inventory (PCDI) scores were used to measure word production and other early language skills. The study measured overall fixation (attention) and looking away (inattention) in terms of duration and frequency for the Single Object Attention and Free Play Task. TTS, CDI, and Single Object Attention and Free Play Tasks were administered at 365 days of age. The ASQ assesses 5 domains: communication, gross motor, fine motor, problem solving, and personal/social, and was conducted at 120, 180, and 275 days of age. Measurements of body weight, length, and head circumference were taken at 30, 90, 120, 180, 275, and 365 days of age. Formula intake, stool characteristics (frequency and consistency) and formula tolerance (fussiness and gassiness) were assessed through a 24-h recall beginning at 90 days of age (Supplementary Table 2). To determine breast milk intake, we utilized data from a survey conducted by the Chinese Center for Disease Control and Prevention on the breast milk intake of Chinese infants aged 0–5 months from 2019 to 2021. The weighing method was employed to measure the 24-hourly breast milk intake of infants. The average breast milk intake of exclusively breastfed infants aged 0–5 months was 800.1 g/d, ranging from 696.4 to 937.7 g/d. Breast milk intake increased with age and remained stable at 5 months (22). Oral, intramuscular and intravenous (IV) antibiotic treatments throughout the study period, and medically-confirmed adverse events (AEs) including respiratory and gastrointestinal infections, were also monitored.

### 2.4 Statistical analysis

The primary outcome for this study is the cognitive scale of the Bayley-III at 365 days of age. Assuming a difference of 6 points and a SD of 15, a sample size of 105 per group is necessary to have a power of 80% when testing at an alpha level of 0.05, two-tailed test. We enrolled 150 participants per group to accommodate a potential attrition rate of 30%. All enrolled participants were included in the analysis. Missing data from participants who withdrew early from the study were not replaced or imputed. Descriptive statistics were used to summarize the continuous variables, including the number of observations, mean, median, standard deviation (SD), and quartiles. The discrete variables were summarized using counts, proportions, and/or percentages. These descriptive analyses were presented separately for each treatment group. A significance level of 0.05 was used for statistical testing, unless otherwise specified. Prior to testing, the distributional assumptions of the outcomes were assessed, and transformations or nonparametric tests were used if necessary. A 95% confidence level was used for confidence intervals. Pairwise group comparisons were conducted for outcome variables that showed significant overall group differences. Tukey adjustment was applied to these comparisons, and adjusted confidence intervals with adjusted *p*-values were reported. All analyses were performed using Version 9.4 of SAS® statistical software.

### 2.5 Intention to treat analysis

Sensitivity analysis was also performed for the primary and secondary outcomes in the intention-to-treat (ITT) population. For outcomes that only observed at post-baseline visits, missing data of early withdrew subjects were handled with multiple imputation with fully conditional specification regression method, based on sex, birth weight, family income, number of family members living in the household, father education, mother education, mother’s age when participant was born, maternal DHA supplement use during last 12 weeks of pregnancy, and maternal prenatal vitamin use during last 12 weeks of pregnancy. Five imputations were conducted for all variables. Group differences were evaluated for each imputed dataset, and the results were combined using the SAS MIANALYZE procedure. For outcomes with longitudinal measurements starting from baseline, we used a mixed model for repeated measures with feeding group, age and their interactions as fixed effect, and an unstructured covariance matrix to compare the differences among feeding groups (23). All outcome data were used, regardless of whether an individual has complete data or not.

## 3 Results

### 3.1 Study population and baseline characteristics

**Figure 1:**
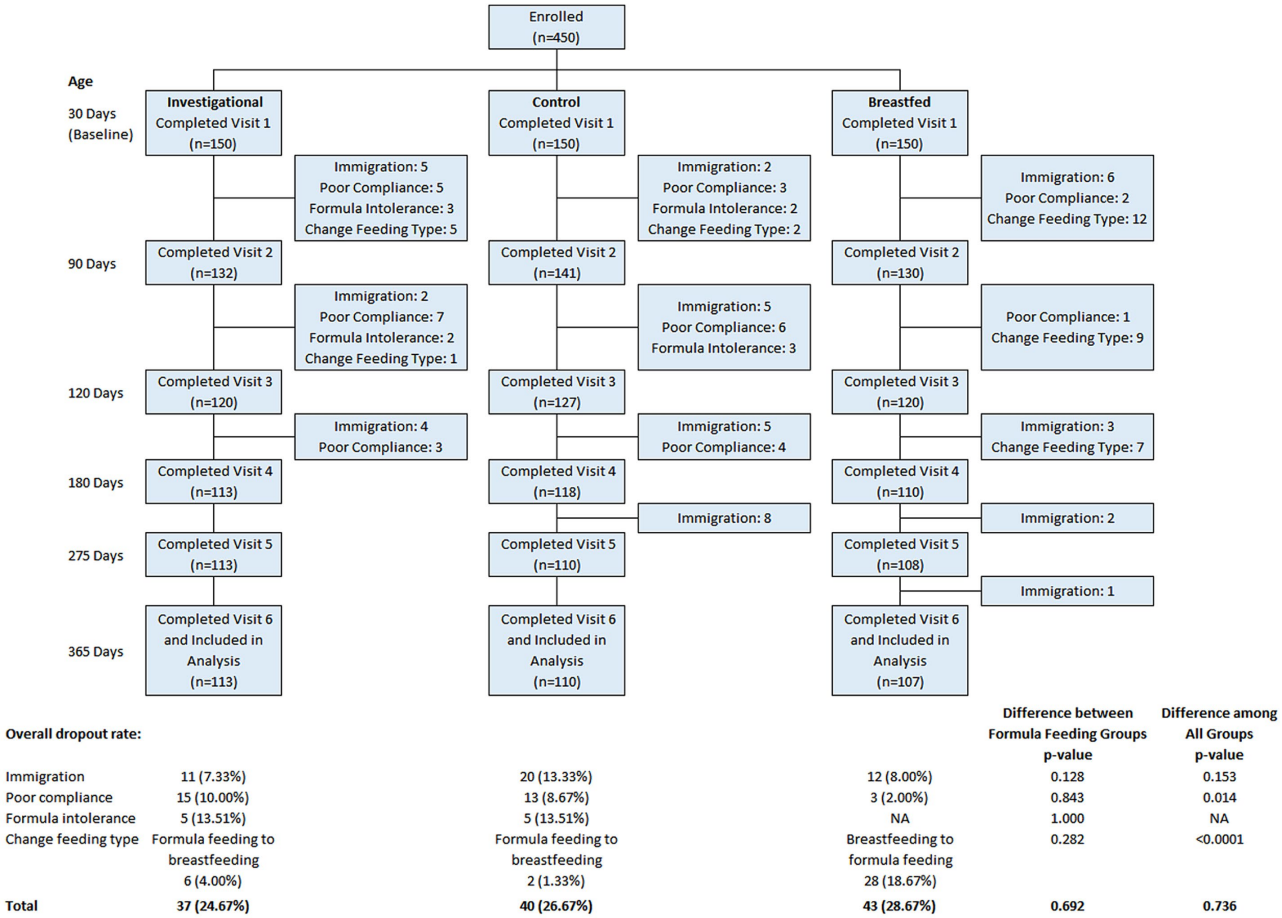
Flow chart of study. Data were presented frequency (%). Group difference was evaluated using chi-square/Fisher’s exact test.

Among the 450 participants recruited, 150 were assigned to the Breastfed group and 300 were randomized to either the Control group (*n* = 150) or the Investigational group (*n* = 150). A total of 330 participants completed the assigned treatment according to study instructions and were included in the analyses. The participant flow from recruitment to study completion is illustrated in Figure 1. There was no significant difference between the formula feeding groups in terms of total discontinuation rate (*p* = 0.692) or the reasons for discontinuation. Table 2 provides a summary of the baseline characteristics for each study group. There were no significant differences in baseline characteristics between the two formula groups. However, the breastfed group had mothers who were younger at the time of participant’s birth, had fewer household members and lower monthly family income compared to the formula groups. Other baseline characteristics were comparable between the breastfed group and the formula groups.

**Table 2:** Baseline characteristics of participants.

Variable	Investigational (*n* = 150)	Control (*n* = 150)	Breastfed (*n* = 150)	Group difference *p*-value
Birth History				
Weight at birth, g	3402.4 ± 372.0	3327.6 ± 372.4	3356.9 ± 380.8	0.221
Length at birth, cm	50.0 ± 0.8	50.0 ± 0.8	49.9 ± 0.8	0.309
Demographics				
Infant age at enrollment, days	33.9 ± 2.2	34.1 ± 2.3	34.1 ± 2.2	0.653
Infant Han ethnic group, %	116 (77.3)	125 (83.3)	112 (74.7)	0.174
Infant boys, %	76 (50.7)	75 (50.0)	75 (50.0)	0.991
Vaginal delivery, %	85 (56.7)	99 (66.0)	97 (64.7)	0.196
Infant gestational age, weeks	39.1 ± 1.0	39.0 ± 1.0	38.9 ± 1.1	0.341
Mother’s age when participant was born, years	30.7 ± 5.1	30.4 ± 4.3	28.6 ± 4.7	**0.0002**
Mother’s marital status, %				0.367
Single	0 (0.0)	0 (0.0)	1 (0.7)	
Married	150 (100.0)	150 (100.0)	149 (99.3)	
Number of previous live births to the infant’s mother				0.698
0	59 (39.3)	50 (33.3)	54 (36.0)	
1	74 (49.3)	86 (57.3)	83 (55.3)	
2	17 (11.3)	13 (8.7)	12 (8.0)	
3	0 (0.0)	1 (0.7)	1 (0.7)	
Infant insurance, %	144 (96.0)	139 (92.7)	141 (94.0)	0.460
Anthropometrics at baseline				
Weight, g	4658.6 ± 520.9	4573.6 ± 489.0	4591.0 ± 497.7	0.303
Length, cm	55.2 ± 1.7	55.0 ± 1.8	55.1 ± 1.8	0.517
Head circumference, cm	37.0 ± 1.0	36.9 ± 1.0	36.9 ± 1.0	0.784
Socioeconomic status				
Number of family members at household	3.5 ± 0.7	3.6 ± 0.6	3.3 ± 0.6	**0.0003**
Household size, %				0.555
<60 m^2^	4 (2.7)	3 (2.0)	7 (4.7)	
60–90 m^2^	83 (55.3)	81 (54.0)	89 (59.3)	
90–120 m^2^	54 (36.0)	59 (39.3)	50 (33.3)	
>120 m^2^	9 (6.0)	7 (4.7)	4 (2.7)	
Mother’s educational level, %				0.872
Primary school	6 (4.0)	6 (4.0)	4 (2.7)	
Junior school	43 (28.7)	43 (28.7)	51 (34.0)	
High school/ Technology school	88 (58.7)	87 (58.0)	82 (54.7)	
Bachelor	13 (8.7)	14 (9.3)	12 (8.0)	
Master and above	0 (0.0)	0 (0.0)	1 (0.7)	
Father’s educational level, %				0.318
Primary school	2 (1.3)	3 (2.0)	5 (3.3)	
Junior school	33 (22.0)	32 (21.3)	45 (30.0)	
High school/ Technology school	84 (56.0)	83 (55.3)	79 (52.7)	
Bachelor	31 (20.7)	32 (21.3)	21 (14.0)	
Master and above	0 (0.0)	0 (0.0)	0 (0.0)	
Mother currently employed, %	87 (58.0)	78 (52.0)	73 (48.7)	0.260
Father currently employed, %	149 (99.3)	147 (98.0)	149 (99.3)	0.626
Monthly average household income, %				**0.001**
<3,000 RMB	0 (0.0)	0 (0.0)	0 (0.0)	
3,000–5,999 RMB	14 (9.3)	19 (12.7)	34 (22.7)	
6,000–8,000 RMB	86 (57.3)	82 (54.7)	94 (62.7)	
>8,000 RMB	50 (33.3)	49 (32.7)	22 (14.7)	
Family medical history				
Infant asthma or allergy history, %	0 (0.0)	0 (0.0)	0 (0.0)	NA
Father asthma or allergy history, %	0 (0.0)	0 (0.0)	0 (0.0)	NA
Mother asthma or allergy history, %	0 (0.0)	0 (0.0)	0 (0.0)	NA
Sibling asthma or allergy history, %	0 (0.0)	0 (0.0)	0 (0.0)	NA
Half-sibling asthma or allergy history, %	0 (0.0)	0 (0.0)	0 (0.0)	NA
Smoker among family members, %	140 (93.3)	140 (93.3)	131 (87.3)	0.103
Exposed in smoking environment, %	3 (2.0)	4 (2.7)	5 (3.3)	0.933
Mother supplement intake within 12 weeks before delivery				
DHA	7 (4.7)	12 (8.0)	11 (7.3)	0.472
Vitamins	33 (22.0)	26 (17.3)	21 (14.0)	0.191

Data presented are mean ± standard deviation for continuous variables and frequency (%) for categorical variables. Group difference was evaluated using one-way analysis of variance for continuous variables and chi-square/Fisher’s exact test for categorical variables.

### 3.2 Primary outcome Bayley-III

The Bayley-III assesses development in five areas: cognitive, language, motor, social–emotional, and adaptive behavior, which were administered at 365 days of age. Considering the baseline differences in characteristics between the breastfed group and the formula milk group. The five developmental scores were analyzed separately using analysis of variance (ANOVA) and using analysis of covariance (ANCOVA) to adjust for potential confounding variables (gender, birth weight, household income, number of family members living in the household, father education, mother education, mother’s age when participant was born, maternal DHA supplement use during last 12 weeks of pregnancy, maternal prenatal vitamin use during last 12 weeks of pregnancy, and exposure to smoking at study enrollment). At 365 days of age, the Bayley-III cognitive composite score was significantly higher for the investigational group compared to the control group (adjusted *p* < 0.0001 for pairwise group comparison, Table 3). The investigational group exhibited significantly higher language (*p* < 0.05) and motor (*p* < 0.05) composite scores compared to the control group. The investigational group and the breastfed group showed similar cognitive and language scores. No significant differences were found between the groups in terms of social–emotional or general adaptive mean scores. These findings remained consistent even after adjusting for factors such as gender, birth weight, family income, parental education, and other socio-environmental variables. In addition, the mean Bayley-III cognitive, language, and motor scores for each group fell within the standardized mean score (mean ± SD = 100 ± 15) of the 50th percentile, indicating mid-average functioning (24). Meanwhile, the results of the ITT analysis were basically consistent with the Per-protocol (PP). Therefore, the study suggests that the infant formula used provided adequate neurodevelopmental support.

**Table 3:** Bayley III composite scores at 365 days of age.

Domain	Investigational	Control	Breastfed	Overall group difference (*p*-value)
***Per-Protocol***	***n* = 113**	***n* = 110**	***n* = 107**	
*Unadjusted*				
Cognitive	104.3 ± 7.7^a^	99.0 ± 5.9^b^	104.7 ± 7.7^a^	**<0.0001**
Language	106.9 ± 5.9^a^	104.5 ± 6.4^b^	107.8 ± 6.7^a^	**0.0002**
Motor	109.2 ± 8.6^a^	103.9 ± 9.5^b^	109.3 ± 12.6^a^	**<0.0001**
Social–emotional	107.7 ± 14.4	105.4 ± 12.9	109.3 ± 15.3	0.136
General adaptive	90.3 ± 11.1	89.7 ± 10.0	91.7 ± 11.3	0.365
*Adjusted* ^1^				
Cognitive	104.3 ± 7.7^a^	99.0 ± 5.9^b^	104.7 ± 7.7^a^	**<0.0001**
Language	106.9 ± 5.9^a^	104.5 ± 6.4^b^	107.8 ± 6.7^a^	**0.0002**
Motor	109.2 ± 8.6^a^	103.9 ± 9.5^b^	109.3 ± 12.6^a^	**<0.0001**
Social–emotional	107.7 ± 14.4	105.4 ± 12.9	109.3 ± 15.3	0.082
General adaptive	90.3 ± 11.1	89.7 ± 10.0	91.7 ± 11.3	0.232
***Intention-To-Treat*** ^2^	***n* = 150**	***n* = 150**	***n* = 150**	
*Unadjusted*				
Cognitive	103.9 ± 9.2^a^	99.8 ± 10.6^b^	104.4 ± 8.8^a^	**<0.0001**
Language	106.7 ± 10.0^a^	105.2 ± 8.8^b^	107.7 ± 6.8^a^	**0.012**
Motor	108.3 ± 13.6^a^	104.7 ± 14.8^b^	108.8 ± 13.5^a^	**0.0003**
Social–emotional	107.8 ± 17.4	105.4 ± 17.1	108.8 ± 16.3	0.240
General adaptive	90.2 ± 15.3	89.7 ± 12.2	91.4 ± 14.6	0.397
*Adjusted* ^1^				
Cognitive	104.3 ± 7.7^a^	99.0 ± 5.9^b^	104.7 ± 7.7^a^	**<0.0001**
Language	106.9 ± 5.9^a^	104.5 ± 6.4^b^	107.8 ± 6.7^a^	**0.002**
Motor	109.2 ± 8.6^a^	103.9 ± 9.5^b^	109.3 ± 12.6^a^	**0.0004**
Social–emotional	107.7 ± 14.4	105.4 ± 12.9	109.3 ± 15.3	0.193
General adaptive	90.3 ± 11.1	89.7 ± 10.0	91.7 ± 11.3	0.244

Data are summarized by mean ± standard deviation. Means with different letters were significantly different (*p* < 0.05).^1^Group differences were analyzed using analysis of covariance, with adjustment for gender, birth weight, family income, number of family members living in the household, father education, mother education, mother’s age when participant was born, maternal DHA supplement use during last 12 weeks of pregnancy, and maternal prenatal vitamin use during last 12 weeks of pregnancy. Tukey adjusted p-values are presented for pair-wise group comparisons.^2^Results are summarized from 5 imputations. Data are summarized by mean ± standard deviation. Group differences were analyzed using mixed model, with adjustment for sex, birth weight, family income, number of family members living in the household, father education, mother education, mother’s age when participant was born, maternal DHA supplement use during last 12 weeks of pregnancy, and maternal prenatal vitamin use during last 12 weeks of pregnancy. Tukey adjusted *p*-values are presented for pair-wise group comparisons.

### 3.3 Secondary outcomes through 12 months of age

The study analyzed the output of the TTS on 9 domains of infant temperament. A significant difference was found in the activity level domain (*p* = 0.007) for TTS scores at 365 days of age. Pairwise comparisons revealed significant differences between the investigational group (*p* < 0.05) and the control (Table 4). However, no statistically significant differences were observed in the other domains of TTS scores. The study measured overall fixation (attention) and looking away (inattention) in terms of duration and frequency for the Single Object Attention and Free Play Task. At 365 days of age, the investigational group had significantly longer total look duration compared to the control group (*p* < 0.05, Table 5). The investigational group and the breastfed group had similar total and mean look duration. There were no significant group differences observed for the longest look duration or the number of look episodes. The ASQ assesses 5 domains: communication, gross motor, fine motor, problem solving, and personal/social. ASQ scores in all five domains were significantly higher in the investigational group compared to the control group at 120 days of age (Supplementary Table 3). The number and percentage of subjects with ASQ scores above, close to or below the cut-off points for each domain are presented in Supplementary Table 4. No significant difference was observed between the investigational group and the control group for the proportion of participants at risk developmentally in all ASQ domains from 120 to 275 days of age. Meanwhile, the ITT analysis results for secondary outcomes were basically consistent with the PP.

**Table 4:** Toddler temperament scale domain scores at 365 days of age.

Domain	Investigational	Control	Breastfed	Overall group difference (*p-*value)
***Per-Protocol*** ^1^	***n* = 113**	***n* = 110**	***n* = 107**	
Activity level	3.8 ± 0.9^a^	3.5 ± 1.0^b^	3.9 ± 0.9^a^	**0.007**
Regularity	3.6 ± 1.3	3.5 ± 1.1	3.6 ± 1.1	0.864
Approach/withdrawal	3.2 ± 1.1	3.2 ± 1.2	3.3 ± 1.2	0.932
Adaptability	3.6 ± 1.1	3.4 ± 0.9	3.6 ± 1.0	0.331
Intensity	3.8 ± 0.8	3.7 ± 0.8	3.8 ± 0.7	0.924
Mood	3.5 ± 1.2	3.4 ± 1.0	3.6 ± 0.9	0.467
Persistence	3.7 ± 1.2	3.6 ± 1.4	3.6 ± 1.2	0.714
Distractibility	4.4 ± 0.9	4.4 ± 1.0	4.3 ± 1.1	0.763
Sensor threshold	3.4 ± 1.3	3.4 ± 1.0	3.4 ± 1.1	0.973
***Intention-To-Treat*** ^2^	***n* = 150**	***n* = 150**	***n* = 150**	
Activity level	3.8 ± 1.0^a^	3.6 ± 1.1^b^	3.8 ± 1.1^a^	**0.042**
Regularity	3.6 ± 1.4	3.5 ± 1.3	3.6 ± 1.4	0.561
Approach/withdrawal	3.2 ± 1.3	3.2 ± 1.3	3.3 ± 1.2	0.674
Adaptability	3.6 ± 1.3	3.4 ± 1.2	3.6 ± 1.1	0.407
Intensity	3.8 ± 0.9	3.8 ± 0.8	3.8 ± 1.0	0.460
Mood	3.5 ± 1.2	3.4 ± 1.1	3.6 ± 1.0	0.408
Persistence	3.7 ± 1.3	3.6 ± 1.7	3.6 ± 1.3	0.532
Distractibility	4.4 ± 1.0	4.4 ± 1.2	4.3 ± 1.3	0.540
Sensor threshold	3.4 ± 1.5	3.4 ± 1.3	3.5 ± 1.3	0.700

Data presented are mean ± standard deviation. Means with different letters were significantly different (*p* < 0.05).^1^Group differences were analyzed using analysis of covariance, with adjustment for gender, birth weight, family income, number of family members living in the household, father education, mother education, mother’s age when participant was born, maternal DHA supplement use during last 12 weeks of pregnancy, and maternal prenatal vitamin use during last 12 weeks of pregnancy. Tukey adjusted p-values are presented for pair-wise group comparisons.^2^Results are summarized from 5 imputations. Data are summarized by mean ± standard deviation. Group differences were analyzed using mixed model, with adjustment for sex, birth weight, family income, number of family members living in the household, father education, mother education, mother’s age when participant was born, maternal DHA supplement use during last 12 weeks of pregnancy, and maternal prenatal vitamin use during last 12 weeks of pregnancy. Tukey adjusted *p*-values are presented for pair-wise group comparisons.

**Table 5:** Five-minute single object free play at 365 days of age.

	Investigational	Control	Breastfed	Overall group difference (*p*-value)
***Per-Protocol*** ^1^	***n* = 113**	***n* = 110**	***n* = 107**	
Look duration				
Longest	58.7 ± 31.9	51.3 ± 39.6	60.6 ± 39.3	0.197
Total	224.7 ± 38.6^a^	201.5 ± 54.7^b^	225.0 ± 43.6^a^	**<0.0001**
Mean	18.1 ± 8.8^ab^	15.8 ± 12.5^b^	21.0 ± 17.5^a^	**0.014**
Look episodes	14.2 ± 4.3	15.0 ± 5.2	14.0 ± 5.2	0.421
***Intention-To-Treat*** ^2^	***n* = 150**	***n* = 150**	***n* = 150**	
Look duration				
Longest	60.6 ± 33.1	54.1 ± 41.4	61.1 ± 40.9	0.296
Total	224.1 ± 42.4^a^	206.0 ± 56.8^b^	222.2 ± 43.6^a^	**0.001**
Mean	18.5 ± 16.2^ab^	18.7 ± 27.7^b^	20.7 ± 18.0^a^	**0.044**
Look episodes	14.1 ± 4.8	14.8 ± 5.7	13.9 ± 5.7	0.384

Data presented are mean ± standard deviation. Means with different letters were significantly different (*p* < 0.05).^1^Group differences were analyzed using analysis of covariance, with adjustment for gender, birth weight, family income, number of family members living in the household, father education, mother education, mother’s age when participant was born, maternal DHA supplement use during last 12 weeks of pregnancy, and maternal prenatal vitamin use during last 12 weeks of pregnancy. Tukey adjusted p-values are presented for pair-wise group comparisons.^2^Results are summarized from 5 imputations. Data are summarized by mean ± standard deviation. Group differences were analyzed using mixed model, with adjustment for sex, birth weight, family income, number of family members living in the household, father education, mother education, mother’s age when participant was born, maternal DHA supplement use during last 12 weeks of pregnancy, and maternal prenatal vitamin use during last 12 weeks of pregnancy. Tukey adjusted *p*-values are presented for pair-wise group comparisons.

### 3.4 Infant growth

Growth rates were analyzed from 30 days to 90, 120, 180, 275 and 365 days of age. No statistically significant group differences by gender were observed in weight, length, or head circumference growth rate (Supplementary Table 5). According to guidance from the American Academy of Pediatrics, the rate of weight gain is a crucial factor to consider when evaluating infant formula clinically. Differences of more than 3 g/day over a 3- to 4-month period are deemed clinically significant (25). The mean weight growth rate of both the control group and the breastfed group did not exceed that of the IF by a clinically relevant amount (3 or more g/day for the 30 to 120-day interval), indicating that the IF provided adequate growth. No statistically significant group differences by gender were observed for mean achieved weight, length or head circumference at any measured time point up to 365 days of age (Supplementary Table 6). Similarly, the results of the ITT showed that both the group effect and the interaction of group and age was not significant, indicating similar patterns of change in achieved weight, length and head circumference over time among the feeding groups (Supplementary Table 7). In addition, the mean achieved weight, length and head circumference for participants on the WHO weight-for-age, length-for-age and head circumference-for-age standard growth chart for boys and girls in each group remained near 50th and 75th percentiles of growth through 365 days of age (Supplementary Figure 1).

### 3.5 Stool characteristics and tolerance

The breastfed group had a significantly higher mean daily stool frequency compared to the investigational group and the control group at 30 days of age. Additionally, the breastfed group had significantly softer stool texture compared to the two formula feeding groups. There were no significant differences in mean daily stool frequency or stool consistency detected between the two formula feeding groups at any visits (Supplementary Table 8). Similarly, the results of the ITT showed that the interaction of group and age was not significant for stool frequency and stool consistency, indicating similar patterns of change in both outcomes over time among the feeding groups. No overall differences were observed between the investigational group and the control group throughout the study period (Supplementary Table 9). Fussiness and amount of gas were similar among the three study groups at all study visits (Supplementary Table 10). Similarly, the results of the ITT showed that the interaction of group and age was not significant for all fussiness and gassiness outcomes, indicating similar patterns of change in tolerance over time among the feeding groups (Supplementary Table 11).

### 3.6 Formula intake and adverse events

The amount of study formula intake was similar between the two formula groups for both boy and girl participants (Supplementary Table 12). In this study, the intake of formula milk fell within the range of breast milk intake, indicating that intake did not affect the study results. The number of subjects AEs was summarized by study group, Medical Dictionary for Regulatory Activities (MedDRA) system organ class and preferred term. There was no significant difference in the rate of each AE type among the study groups (Supplementary Table 13). All AEs reported were of mild intensity and not related to the study products. No serious AEs occurred throughout the study period.

## 4 Discussion

In this study, infants fed an PLs and LCPUFAs supplemented infant formula received significantly higher scores on cognitive testing (Bayley-III) at 12 months of age than did infants fed a control formula. The effect size between *CF* and IF group were reported to be 5.4 for cognitive (104.3 vs. 99.0), 2.4 for language (106.9 vs. 104.5) and 5.3 for motor (109.2 vs. 103.9), respectively.

Previously, RCTs have been carried out to explore cognitive function of either PLs or LCPUFAs for infants. In general, results of PLs (bMFGM) are inconsistent. For example, a Swedish study showed that infant formula supplemented with PLs (bMFGM) significantly increased cognitive level (105.8 vs. 101.8) based on Bayley-III at 1 year of age (14), however, there was no significant difference observed at 6.5 years, with the Breastfeeding Group, used as a reference, showing higher scores in full-scale IQ, verbal comprehension, perceptual reasoning, and working memory from WISC-IV compared to the supplemented group (13). Another study failed to show any effect of PLs (bMFGM) on cognitive level (Bayley-III) of Chinese infants, even though significant improvements were observed for Social emotional and General adaptive scores after adjustment for confounders (15). In terms of LCPUFA, a large number of systematic reviews of RCTs do not support its beneficial role on neurodevelopmental outcomes of infants (26–28). Our results clearly showed that combination of PLs and LCPUFAs support neurodevelopment of infants (three domains were improved), which are robust against a group of known confounders. Notably, the Bayley-III profiles are similar between the IF group and breastfed group (Table 3). Even though direct comparisons are not possible as the infants of breastfed group were not randomized, our exploratory analysis showed that the differences were not significant using the method described in Table 3 (data not shown). Also, it is interesting to note that upon reviewing previous research on LCPUFAs (DHA and ARA) supplementation in infant formula, it was observed that limited studies have explored the specific ratio of ARA to DHA. Despite being often overlooked, this aspect holds significant importance. The balance between ARA and DHA could play a crucial role in influencing cognitive and developmental outcomes during infancy and early childhood. Research on the neurodevelopment of infants who were fed formulas with varying ARA to DHA ratios suggests that the specific ratio utilized may have a substantial impact on the benefits of LC-PUFAs in early childhood, with neurodevelopmental outcomes showing preference for an ARA/DHA ratio of 1:1 or 2:1 (29). Notably, during the initial stages of this clinical trial, researchers meticulously considered the ARA to DHA ratio in the formula design, ultimately settling on a ratio of approximately 1.7. Remarkably, this ratio closely mirrors that found in Chinese breast milk (30). Breast milk is generally considered to be optimal for infant aged 0 ~ 6 months of age, whereby causal relationship has been established between breastfeeding and cognition development (31, 32). It has been recognized in recent years that breast milk represents a complex biological system, such that numerous interacting parts of the system outperforms the sum of those individual parts (33, 34). Therefore, we argue that the neuro-supportive effect of IF in our study may possibly be due to the interplay between PLs and LCPUFA. Gázquez A et al. have demonstrated that incorporating bMFGM plus milk fat into infant formulas may increase the bioavailability of DHA in both plasma and tissues in suckling piglets. The structures of PLs and gangliosides found in bMFGM could potentially enhance the uptake of DHA from milk triacylglycerol and increase its uptake in tissues. The developing brain has the ability to synthesize and incorporate DHA from blood vessels into membrane PLs, which can result in improved neurite outgrowth, synaptogenesis, and neurogenesis (35–37).

To minimize the impact of confounding factors on the results of this study, several potential influencing factors were considered. One of these factors is dietary intake, as higher intake may lead to infants receiving more nutrients and bias the results. Our study specifically examined the intake of formula milk and found no significant difference between the two groups, which was comparable to breastfeeding. Another factor is the addition of dietary supplements for infants, such as supplements containing key components like PLs and LCPUFAs, which may affect the study results. We collected data on dietary supplement usage and found that infants only used supplements containing vitamin D and iron, without including the key components of PLs and LCPUFAs. Additionally, there is limited evidence suggesting that supplementing with omega-3 fatty acids during pregnancy may have a positive impact on cognitive development in children (38). Our study reviewed supplementation during the 12 weeks prior to delivery. Interestingly, we found no significant difference in DHA supplementation among all groups. In addition to diet-related factors, maternal factors may also influence infant cognition. Liu Xiaoning et al. reported that maternal obesity can lead to cognitive and social behavioral deficits in both human and mouse offspring. They also discovered that overweight and obese mothers tend to have lower educational attainment and family income compared to normal-weight mothers (39). Another study found a correlation between economic vulnerability during pregnancy and a higher risk of adverse neurodevelopmental outcomes in 2- and 5-year-old children (40). In our study, we examined the participants’ family income and observed that the monthly income of the breastfeeding group was lower than that of the formula feeding group. However, it is important to note that the monthly income of all participants was above 3,000 yuan, indicating that they were not below the poverty level. Furthermore, we evaluated the education level of the infants’ parents and found no significant difference between the two groups. Unfortunately, we did not track the mothers’ weight during pregnancy. Data from the Children’s Nutrition and Health System Survey in China conducted between 2019 to 2020 suggested that cesarean section delivery was associated with neurodevelopment outcomes. Specifically, it was found that cesarean section might decrease the developmental scores of gross motor, fine motor and language (41). In our study, we did not observe any significant difference in the proportion of mothers’ modes of delivery between the groups. Notably, our research data at each visit were analyzed using analysis of covariance (ANCOVA) to adjust for gender, birth weight, household income, number of family members living in the household, father education, mother education, mother’s age when participant was born, maternal DHA supplement use during last 12 weeks of pregnancy, maternal prenatal vitamin use during last 12 weeks of pregnancy, and exposure to smoking at study enrollment, thereby enhancing the accuracy of result evaluation.

Compared to several previously reported clinical studies (14, 15, 20), our research has the following advantages: a longer feeding period up to 1 year of age for infants, a larger sample size, a more comprehensive neurodevelopmental assessment scale, and a more extensive control group design. However, several limitations of the present study should be noted. Firstly, we did not specifically investigate the content of PLs and LCPUFAs in breast milk in the breastfeeding group. If we had collected such data and compared it to the formula group, there might have been more surprising findings. Secondly, the neurodevelopment of infants is substantially affected by maternal status during pregnancy. However, we only had complete statistics on the occurrence of adverse events during pregnancy, focusing solely on the presence of asthma or allergy history. Thirdly, we did not evaluate the neurodevelopmental aspects of the infants at the time of enrollment, making it impossible to determine whether the effects of innate genetic factors differed between the groups. Nonetheless, it is worth mentioning that our study had a sufficient and randomly distributed sample size, which helps to minimize the influence of confounding factors. Fourthly, human milk oligosaccharides (HMOs) seem to be linked to enhanced white matter development and increased gray matter development in a dose-dependent manner, subsequently leading to improved neurological outcomes during childhood. Various bioactive components influence brain development, particularly myelination, either directly, via the gut-brain axis, or through the immune system (42). HMOs are believed to significantly contribute to long-term cognitive improvement. However, the study did not yield any significant findings regarding the impact of HMOs on the microbiota in the two experimental groups. Fifthly, it is unfortunate that this clinical study did not incorporate relevant blood measurements, particularly the levels of DHA in red blood cell membrane PLs, which indicates the level of DHA in cerebral cell membranes. The inclusion of this data would have further strengthened the evidence supporting the hypothesis that LCPUFA and PLs work together to produce neurodevelopmental effects. Lastly, it is important to highlight that our study only reported short-term results within the first year of birth, and further investigation is needed to assess long-term outcomes. It is worth noting that in two other studies, the investigational group (bMFGM + DHA + ARA) demonstrated better cognitive scores at 18 months, 30 months, and 4 years of age (43, 44).

In conclusion, the study found that the infant formula, which included PLs and LCPUFAs are safe, tolerated which support normal growth in infants. More importantly, infants fed the PLs and LCPUFAs supplemented infant formula performed significantly better (including cognitive, language, and motor skills, as well as longer sustained attention) on cognitive testing at 12 months of age than did infants fed a control formula. Further research is needed to understand any potential long-term effects on growth and neurodevelopment due to possible programming and interacting effects of PLs and LCPUFAs.

## Data availability statement

The original contributions presented in the study are included in the article/Supplementary material, further inquiries can be directed to the corresponding authors.

## Ethics statement

The studies involving humans were approved by Institutional Review Board of Shanghai Nutrition Society. The studies were conducted in accordance with the local legislation and institutional requirements. Written informed consent for participation in this study was provided by the participants’ legal guardians/next of kin.

## Author contributions

QR: Formal analysis, Investigation, Writing – original draft. XZ: Investigation, Writing – review & editing. JP: Investigation, Resources, Writing – review & editing. KL: Formal analysis, Writing – original draft. YZ: Resources, Writing – review & editing. YL: Formal analysis, Writing – review & editing. QX: Conceptualization, Writing – original draft, Writing – review & editing. YX: Conceptualization, Supervision, Writing – review & editing.
