# Supplementary material for: A combination of phospholipids and long chain polyunsaturated fatty acids supports neurodevelopmental outcomes in infants: a randomized, double-blind, controlled clinical trial
Source: Front Nutr. 2024 Jun 13;11:1358651. doi: 10.3389/fnut.2024.1358651 (PMC11208465; doi:10.3389/fnut.2024.1358651)
Supplement: Supplementary file 1 [file Data_Sheet_1.docx]

Supplementary Material

# Supplementary Figures and Tables

SUPPLEMENTARY TABLE 1 Data collection.

| **Item** | Visit 1  30 (±7) days of age, enrollment | Visit 2  90 (±7) days of age | Visit 3  120 (±7) days of age | Visit 4  180 (±7) days of age | Visit 5  275 (±7) days of age | Visit 6  365 (±7) days of age |
| --- | --- | --- | --- | --- | --- | --- |
| Informed consent | √ |  |  |  |  |  |
| Participant Inclusion Criteria | √ |  |  |  |  |  |
| Participant medical records | √ |  |  |  |  |  |
| Demographics (Infant age, race, gender, delivery mode, gestational age, mother’s age when participant was born, mother’s marital status, number of previous live births to the infant’s mother) | √ |  |  |  |  |  |
| Socioeconomic status (Number of family members, household size, parent’s educational level, parent currently employed, household income) | √ |  |  |  |  |  |
| Family medical history, smoking situation, mother supplement intake within 12 weeks before delivery (DHA, Vitamin) | √ |  |  |  |  |  |
| Anthropometrics (weight, length, and head circumference) | √ | √ | √ | √ | √ | √ |
| Formula intake, Stool characteristics and formula tolerance (24-hour recall) |  | √ | √ | √ | √ | √ |
| Infant dietary supplement records |  |  | √ | √ | √ | √ |
| Antibiotic treatment | √ | √ | √ | √ | √ | √ |
| Bayley-III, TTS, CDI, Single Object Attention and Free Play Tasks |  |  |  |  |  | √ |
| ASQ |  |  | √ | √ | √ |  |
| Medically-confirmed adverse events | √ | √ | √ | √ | √ | √ |

**SUPPLEMENTARY TABLE 2** Questionnaires on formula intake, stool characteristics, and tolerance (24-hour recall).

| **Table ID Random number of volunteers Abbreviation of volunteer name**  _______ _______________________ _______________________  **Formula intake, stool characteristics, and tolerance (24-hour recall) - visit 2**  Date of filling in the form: □□□□/□□/□□  **Visit 2 (90 days ± 7 days)**  1. How much formula has your baby taken in the past 24 hours? ________ ml of liquid   1. Since the last visit, has your baby taken other formula or milk? (single choice)   □ No □ Yes  If "Yes", how much do your baby take other formula or milk per day? ________ml of liquid. How many days do your baby take other formula or milk? ____days  3. How many times did your baby defecate in the past 24 hours? _____  4. What is the usual stool consistency of your baby in the past 24 hours? (single choice)  0 □ No defecation  1 □ Hard - dry, hard and lumpy  2 □ Formed - formed, not dry  3 □ Soft - unformed, muddy  4 □ Unformed or seedy (not formed, with water or small particles)  5 □ Watery - unformed, watery  5. Fussiness in the past 24 hours? (single choice)  0 □ Not at all  1 □ Slightly  2 □ Moderately  3 □ Very  4 □ Extremely  6. Fussiness in the past 24 hours, your baby: (single choice)  0 □ Less than usual  1 □ Same as usual  2 □ More than usual  7. Gassiness in the past 24 hours? (single choice)  0 □ Not at all  1 □ Slight amount  2 □ Moderate amount  3 □ Excessive amount  8. Gassiness in the past 24 hours, your baby: (single choice)  0 □ Less than usual  1 □ Same as usual  2 □ More than usual |
| --- |

**SUPPLEMENTARY TABLE 3** Ages and stages questionnaire domain score from 120 to 275 days of age.

| **Age** | **Domain** | **Investigational** | **Control** | **Breastfed** | **Overall Group Difference (p-value)** |
| --- | --- | --- | --- | --- | --- |
| ***Per-Protocol*** ^1^ | |  |  |  |  |
| 120 days | N | 120 | 127 | 120 |  |
|  | Communication | 52.5±7.0 ^a^ | 50.2±7.2 ^b^ | 52.7±6.8 ^a^ | **0.018** |
|  | Gross motor | 53.6±6.7 ^a^ | 51.1±8.1 ^b^ | 53.8±6.4 ^a^ | **0.011** |
|  | Fine motor | 51.3±8.8 ^a^ | 48.5±9.9 ^b^ | 51.8±8.3 ^a^ | **0.006** |
|  | Problem solving | 53.6±6.7 ^a^ | 51.3±6.9 ^b^ | 53.8±6.8 ^a^ | **0.009** |
|  | Personal/social | 51.7±7.4 ^a^ | 48.7±7.2 ^b^ | 52.0±8.0 ^a^ | **0.001** |
| 180 days | N | 113 | 118 | 110 |  |
|  | Communication | 52.8±5.0 | 51.4±6.6 | 53.0±5.8 | 0.143 |
|  | Gross motor | 50.7±9.2 | 48.1±9.5 | 51.2±8.6 | 0.073 |
|  | Fine motor | 54.7±7.0 | 53.1±8.4 | 55.0±7.6 | 0.062 |
|  | Problem solving | 53.7±6.7 | 51.8±8.2 | 53.9±7.2 | 0.087 |
|  | Personal/social | 51.1±8.6 | 49.5±9.9 | 51.6±8.5 | 0.145 |
| 275 days | N | 113 | 110 | 108 |  |
|  | Communication | 53.6±7.3 | 52.6±7.4 | 53.8±6.8 | 0.447 |
|  | Gross motor | 47.4±8.7 | 46.0±8.4 | 48.0±7.2 | 0.422 |
|  | Fine motor | 55.8±5.9 | 54.8±7.5 | 56.0±6.2 | 0.258 |
|  | Problem solving | 54.5±7.41 | 54.1±7.9 | 55.0±6.7 | 0.891 |
|  | Personal/social | 53.2±7.68 | 52.2±8.7 | 53.3±7.8 | 0.376 |
| ***Intention-To-Treat*** ^2^ | | |  |  |  |
| 120 days | N | 150 | 150 | 150 |  |
|  | Communication | 52.0±7.7 ^a^ | 50.1±7.3 ^b^ | 52.2±7.7 ^a^ | **0.037** |
|  | Gross motor | 53.2±6.9 ^a^ | 51.1±7.9 ^b^ | 53.3±6.9 ^a^ | **0.024** |
|  | Fine motor | 50.8±8.9 ^a^ | 48.5±9.7 ^b^ | 51.1±9.0 ^a^ | **0.014** |
|  | Problem solving | 53.1±7.1 ^a^ | 51.2±7.9 ^b^ | 53.1±7.2 ^a^ | **0.040** |
|  | Personal/social | 50.9±7.4 ^a^ | 48.7±7.4 ^b^ | 51.5±8.2 ^a^ | **0.003** |
| 180 days | N | 150 | 150 | 150 |  |
|  | Communication | 52.7±5.8 | 51.5±7.1 | 52.7±6.1 | 0.234 |
|  | Gross motor | 50.3±10.5 | 48.0±10.4 | 50.7±9.4 | 0.116 |
|  | Fine motor | 54.7±8.8 | 53.0±9.3 | 54.8±8.7 | 0.120 |
|  | Problem solving | 53.4±7.3 | 51.8±8.8 | 53.3±7.5 | 0.195 |
|  | Personal/social | 50.8±10.6 | 49.5±10.5 | 51.3±9.6 | 0.233 |
| 275 days | N | 150 | 150 | 150 |  |
|  | Communication | 53.2±7.8 | 52.2±8.3 | 53.3±7.3 | 0.318 |
|  | Gross motor | 47.1±9.9 | 45.4±9.0 | 47.6±8.3 | 0.292 |
|  | Fine motor | 55.7±6.8 | 54.5±7.7 | 56.0±8.2 | 0.268 |
|  | Problem solving | 54.4±7.5 | 54.1±7.9 | 54.8±8.8 | 0.669 |
|  | Personal/social | 53.0±8.7 | 51.9±9.1 | 53.1±8.7 | 0.239 |

Data presented are mean ± standard deviation. Means with different letters were significantly different (P < 0.05). ^a^ no significant difference between investigational and breastfed; ^b^ significant difference between investigational and control, and significant difference between control and breastfed.

^1^ Group differences were analyzed using analysis of covariance, with adjustment for gender, birth weight, family income, number of family members living in the household, father education, mother education, mother’s age when participant was born, maternal DHA supplement use during last 12 weeks of pregnancy, and maternal prenatal vitamin use during last 12 weeks of pregnancy. Tukey adjusted p-values are presented for pair-wise group comparisons.

^2^ Results are summarized from 5 imputations. Data are summarized by mean ± standard deviation. Group differences were analyzed using mixed model, with adjustment for sex, birth weight, family income, number of family members living in the household, father education, mother education, mother’s age when participant was born, maternal DHA supplement use during last 12 weeks of pregnancy, and maternal prenatal vitamin use during last 12 weeks of pregnancy. Tukey adjusted p-values are presented for pair-wise group comparisons.

**SUPPLEMENTARY TABLE 4** Participants at risk developmentally by ages and stages questionnaire domain score from 120 to 275 days of age

| **Age** | **Domain** | **Compare to Cutoff** | **Investigational** | **Control** | **Breastfed** | **Overall Group Difference (p-value)** |
| --- | --- | --- | --- | --- | --- | --- |
| 120 days | N |  | 120 | 127 | 120 |  |
|  | Communication | Above | 111 (92.5) | 105 (82.7) | 113 (94.2) | **0.014** |
|  |  | Close to | 9 (7.5) | 20 (15.8) | 6 (5.0) |  |
|  |  | Below | 0 (0.0) | 2 (1.6) | 1 (0.8) |  |
|  | Gross motor | Above | 99 (82.5) | 92 (72.4) | 106 (88.3) | **0.008** |
|  |  | Close to | 21 (17.5) | 32 (25.2) | 13 (10.8) |  |
|  |  | Below | 0 (0.0) | 3 (2.4) | 1 (0.8) |  |
|  | Fine motor | Above | 101 (84.2) | 89 (70.1) | 105 (87.5) | **0.004** |
|  |  | Close to | 18 (15.0) | 36 (28.4) | 14 (11.7) |  |
|  |  | Below | 1 (0.8) | 2 (1.6) | 1 (0.8) |  |
|  | Problem solving | Above | 110 (91.7) | 110 (86.6) | 110 (91.7) | 0.339 |
|  |  | Close to | 10 (8.3) | 17 (13.4) | 10 (8.3) |  |
|  |  | Below | 0 (0.0) | 0 (0.0) | 0 (0.0) |  |
|  | Personal/social | Above | 109 (90.8) | 102 (80.3) | 106 (88.3) | 0.097 |
|  |  | Close to | 9 (7.5) | 23 (18.1) | 13 (10.8) |  |
|  |  | Below | 2 (1.7) | 2 (1.6) | 1 (0.8) |  |
| 180 days | N |  | 113 | 118 | 110 |  |
|  | Communication | Above | 112 (99.1) | 115 (97.5) | 110 (100.0) | 0.330 |
|  |  | Close to | 1 (0.9) | 3 (2.5) | 0 (0.0) |  |
|  |  | Below | 0 (0.0) | 0 (0.0) | 0 (0.0) |  |
|  | Gross motor | Above | 105 (92.9) | 109 (91.6) | 104 (94.6) | 0.639 |
|  |  | Close to | 7 (6.2) | 6 (5.0) | 5 (4.6) |  |
|  |  | Below | 1 (0.9) | 4 (3.4) | 1 (0.9) |  |
|  | Fine motor | Above | 111 (98.2) | 114 (95.8) | 107 (97.3) | 0.889 |
|  |  | Close to | 2 (1.8) | 4 (3.4) | 3 (2.7) |  |
|  |  | Below | 0 (0.0) | 1 (0.8) | 0 (0.0) |  |
|  | Problem solving | Above | 111 (98.2) | 113 (95.0) | 108 (98.2) | 0.362 |
|  |  | Close to | 2 (1.8) | 6 (5.0) | 2 (1.8) |  |
|  |  | Below | 0 (0.0) | 0 (0.0) | 0 (0.0) |  |
|  | Personal/social | Above | 103 (91.2) | 105 (88.2) | 100 (90.9) | 0.806 |
|  |  | Close to | 8 (7.1) | 10 (8.4) | 9 (8.2) |  |
|  |  | Below | 2 (1.8) | 4 (3.4) | 1 (0.9) |  |
| 275 cays | N |  | 113 | 110 | 108 |  |
|  | Communication | Above | 110 (97.4) | 107 (97.3) | 108 (100.0) | 0.254 |
|  |  | Close to | 3 (2.7) | 3 (2.7) | 0 (0.0) |  |
|  |  | Below | 0 (0.0) | 1 (0.9) | 0 (0.0) |  |
|  | Gross motor | Above | 106 (93.8) | 101 (91.8) | 104 (96.3) | 0.532 |
|  |  | Close to | 7 (6.2) | 8 (7.3) | 4 (3.7) |  |
|  |  | Below | 0 (0.0) | 1 (0.9) | 0 (0.0) |  |
|  | Fine motor | Above | 110 (97.4) | 100 (90.9) | 103 (95.4) | 0.167 |
|  |  | Close to | 2 (2.7) | 9 (8.2) | 5 (4.6) |  |
|  |  | Below | 0 (0.0) | 1 (0.9) | 0 (0.0) |  |
|  | Problem solving | Above | 111 (98.2) | 106 (96.4) | 106 (98.2) | 0.671 |
|  |  | Close to | 2 (1.8) | 4 (3.6) | 2 (1.9) |  |
|  |  | Below | 0 (0.0) | 0 (0.0) | 0 (0.0) |  |
|  | Personal/social | Above | 110 (97.4) | 105 (95.5) | 106 (98.2) | 0.527 |
|  |  | Close to | 3 (2.7) | 5 (4.6) | 2 (1.9) |  |
|  |  | Below | 0 (0.0) | 0 (0.0) | 0 (0.0) |  |

Data presented are frequency (percentage). Group differences were evaluated using Fisher’s exact test.

**SUPPLEMENTARY TABLE 5** Weight, length and head circumference growth rates by gender from 30 days to 90 and 120 days of age

| **Age** | **Outcome** | **Investigational** | **Control** | **Breastfed** | **Overall Group Difference (p-value)** |
| --- | --- | --- | --- | --- | --- |
| ***Boys*** |  |  |  |  |  |
| 90 days | N | 65 | 69 | 68 |  |
|  | Weight, g/day | 36.2±10.0 | 36.1±10.0 | 36.2±9.3 | 0.986 |
|  | Length, cm/day | 0.11±0.028 | 0.12±0.023 | 0.11±0.024 | 0.889 |
|  | HC, cm/day | 0.05±0.016 | 0.05±0.014 | 0.05±0.017 | 0.925 |
| 120 days | N | 57 | 63 | 64 |  |
|  | Weight, g/day | 29.8±6.6 | 29.8±6.6 | 29.8±6.8 | 0.974 |
|  | Length, cm/day | 0.11±0.018 | 0.10±0.018 | 0.11±0.016 | 0.710 |
|  | HC, cm/day | 0.05±0.011 | 0.05±0.011 | 0.05±0.011 | 0.852 |
| 180 days | N | 53 | 61 | 59 |  |
|  | Weight, g/day | 25.2±4.7 | 24.6±4.7 | 24.7±4.2 | 0.724 |
|  | Length, cm/day | 0.09±0.01 | 0.09±0.01 | 0.09±0.01 | 0.857 |
|  | HC, cm/day | 0.04±0.001 | 0.04±0.01 | 0.04±0.01 | 0.805 |
| 275 days | N | 53 | 60 | 57 |  |
|  | Weight, g/day | 19.0±3.3 | 18.8±3.2 | 18.9±2.8 | 0.903 |
|  | Length, cm/day | 0.07±0.01 | 0.07±0.01 | 0.07±0.01 | 0.695 |
|  | HC, cm/day | 0.03±0.005 | 0.03±0.004 | 0.03±0.004 | 0.529 |
| 365 days | N | 53 | 60 | 57 |  |
|  | Weight, g/day | 16.4±2.6 | 16.3±2.9 | 16.3±2.2 | 0.962 |
|  | Length, cm/day | 0.06±0.01 | 0.06±0.01 | 0.06±0.01 | 0.701 |
|  | HC, cm/day | 0.03±0.004 | 0.03±0.003 | 0.03±0.004 | 0.269 |
| ***Girls*** |  |  |  |  |  |
| 90 days | N | 67 | 72 | 62 |  |
|  | Weight, g/day | 30.7±8.8 | 30.5±8.1 | 30.6±8.8 | 0.983 |
|  | Length, cm/day | 0.11±0.020 | 0.10±0.026 | 0.10±0.025 | 0.721 |
|  | HC, cm/day | 0.05±0.017 | 0.05±0.016 | 0.05±0.016 | 0.547 |
| 120 days | N | 63 | 64 | 56 |  |
|  | Weight, g/day | 25.6±5.4 | 25.5±5.7 | 25.6±5.8 | 0.911 |
|  | Length, cm/day | 0.10±0.016 | 0.10±0.017 | 0.10±0.016 | 0.862 |
|  | HC, cm/day | 0.04±0.011 | 0.04±0.009 | 0.04±0.011 | 0.535 |
| 180 days | N | 60 | 57 | 51 |  |
|  | Weight, g/day | 21.7±4.1 | 21.8±4.8 | 21.8±4.0 | 0.989 |
|  | Length, cm/day | 0.08±0.01 | 0.08±0.01 | 0.08±0.01 | 0.830 |
|  | HC, cm/day | 0.04±0.01 | 0.04±0.01 | 0.04±0.01 | 0.970 |
| 275 days | N | 60 | 50 | 51 |  |
|  | Weight, g/day | 17.0±2.6 | 16.9±2.5 | 16.9±3.0 | 0.837 |
|  | Length, cm/day | 0.06±0.01 | 0.07±0.01 | 0.06±0.01 | 0.887 |
|  | HC, cm/day | 0.03±0.005 | 0.03±0.004 | 0.03±0.004 | 0.642 |
| 365 days | N | 60 | 50 | 50 |  |
|  | Weight, g/day | 14.8±2.1 | 14.9±2.5 | 14.8±2.0 | 0.967 |
|  | Length, cm/day | 0.06±0.01 | 0.06±0.01 | 0.06±0.01 | 0.839 |
|  | HC, cm/day | 0.03±0.003 | 0.03±0.003 | 0.03±0.003 | 0.919 |

Data presented are mean ± standard deviation. Group differences were analyzed using analysis of covariance, with adjustment of baseline anthropometric measures.

**SUPPLEMENTARY TABLE 6** Achieved weight, length and head circumference by gender at 30, 90, 120, 180, 275 and 365 days of age

| **Age** | **Outcome** | **Investigational** | **Control** | **Breastfed** | **Overall Group Difference (p-value)** |
| --- | --- | --- | --- | --- | --- |
|  |  |  |  |  |  |
| ***Boys*** |  |  |  |  |  |
| 30 days | N | 76 | 75 | 75 |  |
|  | Weight, g | 4754±523 | 4686±542 | 4678±534 | 0.633 |
|  | Length, cm | 55.6±1.7 | 55.7±1.8 | 55.4±1.8 | 0.503 |
|  | HC, cm | 37.3±1.0 | 37.3±1.0 | 37.2±1.0 | 0.771 |
| 90 days | N | 65 | 69 | 68 |  |
|  | Weight, g | 6940±770 | 6826±742 | 6841±678 | 0.938 |
|  | Length, cm | 62.5±1.9 | 62.4±1.8 | 62.1±2.2 | 0.848 |
|  | HC, cm | 40.4±1.0 | 40.3±1.0 | 40.2±1.2 | 0.860 |
| 120 days | N | 57 | 63 | 64 |  |
|  | Weight, g | 7426±766 | 7301±743 | 7358±688 | 0.890 |
|  | Length, cm | 65.1±2.0 | 64.8±1.8 | 64.9±1.8 | 0.486 |
|  | HC, cm | 41.4±0.9 | 41.3±1.0 | 41.3±1.1 | 0.722 |
| 180 days | N | 53 | 61 | 59 |  |
|  | Weight, g | 8467±861 | 8318±861 | 8373±725 | 0.806 |
|  | Length, cm | 68.6±2.1 | 68.6±2.1 | 68.5±2.1 | 0.940 |
|  | HC, cm | 43.3±1.1 | 43.2±1.2 | 43.3±1.1 | 0.883 |
| 275 days | N | 53 | 60 | 57 |  |
|  | Weight, g | 9344±945 | 9223±891 | 9296±728 | 0.902 |
|  | Length, cm | 72.1±2.1 | 71.9±2.2 | 71.9±2.2 | 0.699 |
|  | HC, cm | 44.9±1.1 | 44.7±1.2 | 44.8±1.0 | 0.533 |
| 365 days | N | 53 | 60 | 57 |  |
|  | Weight, g | 10158±973 | 10075±966 | 10108±789 | 0.972 |
|  | Length, cm | 75.8±2.1 | 75.6±1.9 | 75.7±1.9 | 0.768 |
|  | HC, cm | 46.1±1.1 | 45.9±1.1 | 46.1±1.0 | 0.294 |
| ***Girls*** |  |  |  |  |  |
| 30 days | N | 74 | 75 | 75 |  |
|  | Weight, g | 4561±503 | 4461±402 | 4504±444 | 0.402 |
|  | Length, cm | 54.9±1.5 | 54.3±1.6 | 54.8±1.7 | 0.096 |
|  | HC, cm | 36.7±1.0 | 36.6±0.8 | 36.6±0.9 | 0.680 |
| 90 days | N | 67 | 72 | 62 |  |
|  | Weight, g | 6404±685 | 6288±582 | 6371±697 | 0.960 |
|  | Length, cm | 61.2±1.8 | 60.5±1.8 | 61.0±2.1 | 0.792 |
|  | HC, cm | 39.6±1.1 | 39.4±1.1 | 39.5±1.0 | 0.625 |
| 120 days | N | 63 | 64 | 56 |  |
|  | Weight, g | 6871±661 | 6743±589 | 6807±642 | 0.907 |
|  | Length, cm | 63.6±1.8 | 63.0±1.4 | 63.4±2.0 | 0.878 |
|  | HC, cm | 40.6±1.0 | 40.3±0.8 | 40.5±1.1 | 0.488 |
| 180 days | N | 60 | 57 | 51 |  |
|  | Weight, g | 7817±713 | 7689±769 | 7762±725 | 0.959 |
|  | Length, cm | 67.2±1.8 | 66.5±1.5 | 66.9±2.0 | 0.737 |
|  | HC, cm | 42.4±1.2 | 42.2±1.2 | 42.3±1.3 | 0.936 |
| 275 days | N | 60 | 50 | 51 |  |
|  | Weight, g | 8721±675 | 8550±663 | 8622±842 | 0.806 |
|  | Length, cm | 70.6±1.9 | 70.2±2.0 | 70.4±2.2 | 0.835 |
|  | HC, cm | 43.9±1.1 | 43.7±0.9 | 43.9±1.0 | 0.669 |
| 365 days | N | 60 | 50 | 50 |  |
|  | Weight, g | 9513±743 | 9409±814 | 9424±773 | 0.959 |
|  | Length, cm | 74.5±2.0 | 74.2±2.0 | 74.2±2.5 | 0.799 |
|  | HC, cm | 45.1±1.0 | 44.9±1.0 | 45.0±1.1 | 0.949 |

Data presented are mean ± standard deviation. Group differences at 30 days of age were analyzed using analysis of variance. Group differences from 90 to 365 days of age were analyzed using analysis of covariance, with adjustment of baseline anthropometric measures.

**SUPPLEMENTARY TABLE 7** Group comparisons by gender in achieved weight, length and head circumference throughout the study from 30 to 365 days of age

| **Gender** | **Outcome** | **Model Effect (p-value)** | | |
| --- | --- | --- | --- | --- |
|  |  | **Group** | **Age** | **Group*Age Interaction** |
| ***Boys*** | Weight, g | 0.784 | <0.0001 | 0.999 |
|  | Length, cm | 0.754 | <0.0001 | 0.986 |
|  | HC, cm | 0.825 | <0.0001 | 0.965 |
| ***Girls*** | Weight, g | 0.617 | <0.0001 | 0.999 |
|  | Length, cm | 0.230 | <0.0001 | 0.807 |
|  | HC, cm | 0.421 | <0.0001 | 0.981 |

Group differences throughout the study period from 30 to 365 days of age were analyzed using mixed models.

**SUPPLEMENTARY TABLE 8** Stool characteristics in the past 24 hours of each visit

| **Visit (Age)** | **Outcome** | **Investigational** | **Control** | **Breastfed** | **Overall Group Difference (p-value)** |
| --- | --- | --- | --- | --- | --- |
| 30 days | N | 150 | 150 | 150 |  |
|  | Stool frequency | 2.1±0.6 | 2.1±0.6 | 2.4±0.8 | **0.001** |
|  | Stool consistency |  |  |  | **0.012** |
|  | 0=No defecation | 2 (1.33) | 2 (1.33) | 1 (0.67) |  |
|  | 1=Hard | 0 (0.00) | 1 (0.67) | 1 (0.67) |  |
|  | 2=Formed | 19 (12.67) | 23 (15.33) | 12 (8.00) |  |
|  | 3=Soft | 106 (70.67) | 104 (69.33) | 100 (66.67) |  |
|  | 4=Unformed or seedy | 23 (15.33) | 20 (13.33) | 36 (24.00) |  |
|  | 5=Watery | 0 (0.00) | 0 (0.00) | 0 (0.00) |  |
| 90 days | N | 132 | 141 | 130 |  |
|  | Stool frequency | 1.4±0.5 | 1.3±0.6 | 1.5±0.7 | 0.098 |
|  | Stool consistency |  |  |  | 0.102 |
|  | 0=No defecation | 5 (3.79) | 3 (2.13) | 4 (3.08) |  |
|  | 1=Hard | 0 (0.00) | 0 (0.00) | 1 (0.77) |  |
|  | 2=Formed | 24 (18.18) | 41 (29.08) | 8 (6.15) |  |
|  | 3=Soft | 98 (74.24) | 94 (66.67) | 111 (85.38) |  |
|  | 4=Unformed or seedy | 5 (3.79) | 3 (2.13) | 5 (3.85) |  |
|  | 5=Watery | 0 (0.00) | 0 (0.00) | 1 (0.77) |  |
| 120 days | N | 120 | 127 | 120 |  |
|  | Stool frequency | 1.2±0.4 | 1.2±0.5 | 1.4±0.5 | 0.078 |
|  | Stool consistency |  |  |  | 0.303 |
|  | 0=No defecation | 0 (0.00) | 2 (1.57) | 1 (0.83) |  |
|  | 1=Hard | 0 (0.00) | 3 (2.36) | 0 (0.00) |  |
|  | 2=Formed | 32 (26.67) | 36 (28.35) | 36 (30.00) |  |
|  | 3=Soft | 85 (70.83) | 83 (65.35) | 79 (65.83) |  |
|  | 4=Unformed or seedy | 3 (2.50) | 3 (2.36) | 4 (3.33) |  |
|  | 5=Watery | 0 (0.00) | 0 (0.00) | 0 (0.00) |  |
| 180 days | N | 113 | 118 | 110 |  |
|  | Stool frequency | 1.2±0.5 | 1.2±0.5 | 1.3±0.6 | 0.230 |
|  | Stool consistency |  |  |  | 0.290 |
|  | 0=No defecation | 3 (2.65) | 6 (5.08) | 4 (3.64) |  |
|  | 1=Hard | 0 (0.00) | 1 (0.85) | 0 (0.00) |  |
|  | 2=Formed | 44 (38.94) | 49 (41.53) | 37 (33.64) |  |
|  | 3=Soft | 66 (58.41) | 61 (51.69) | 68 (61.82) |  |
|  | 4=Unformed or seedy | 0 (0.00) | 1 (0.85) | 1 (0.91) |  |
|  | 5=Watery | 0 (0.00) | 0 (0.00) | 0 (0.00) |  |
| 275 days | N | 113 | 110 | 108 |  |
|  | Stool frequency | 1.2±0.4 | 1.1±0.4 | 1.3±0.5 | 0.104 |
|  | Stool consistency |  |  |  | 0.292 |
|  | 0=No defecation | 3 (2.65) | 5 (4.55) | 2 (1.85) |  |
|  | 1=Hard | 0 (0.00) | 2 (1.82) | 2 (1.85) |  |
|  | 2=Formed | 81 (71.68) | 78 (70.91) | 72 (66.67) |  |
|  | 3=Soft | 28 (24.78) | 25 (22.73) | 32 (29.63) |  |
|  | 4=Unformed or seedy | 1 (0.88) | 0 (0.00) | 0 (0.00) |  |
|  | 5=Watery | 0 (0.00) | 0 (0.00) | 0 (0.00) |  |
| 365 days | N | 113 | 110 | 107 |  |
|  | Stool frequency | 1.2±0.5 | 1.1±0.5 | 1.2±0.5 | 0.475 |
|  | Stool consistency |  |  |  | 0.224 |
|  | 0=No defecation | 2 (1.77) | 4 (3.64) | 3 (2.80) |  |
|  | 1=Hard | 3 (2.65) | 6 (5.45) | 1 (0.93) |  |
|  | 2=Formed | 103 (91.15) | 96 (87.27) | 96 (89.72) |  |
|  | 3=Soft | 5 (4.42) | 4 (3.64) | 6 (5.61) |  |
|  | 4=Unformed or seedy | 0 (0.00) | 0 (0.00) | 1 (0.93) |  |
|  | 5=Watery | 0 (0.00) | 0 (0.00) | 0 (0.00) |  |

Data presented are mean ± standard deviation or count (%). Group differences were analyzed using analysis of variance for continuous outcomes and Cochran-Mantel-Haenszel row-mean score test for ordinal outcomes.

**SUPPLEMENTARY TABLE 9** Group comparisons of stool characteristics in the past 24 hours throughout the study from 30 to 365 days of age and each visit (Mixed model analysis)

| **Outcome** | **Age** | **Model Effect (p-value)** | | | **Pairwise Group Comparison**  **(Adjusted p-value)** | | |
| --- | --- | --- | --- | --- | --- | --- | --- |
|  |  | **Group** | **Age** | **Group*Age Interaction** | **Investigational vs. Control** | **Investigational vs. Breastfed** | **Control vs. Breastfed** |
| Stool frequency ^1^ | Overall | <0.0001 | <0.0001 | 0.786 | 0.761 | 0.0001 | <0.0001 |
|  | 30 days |  |  |  | 1.000 | 0.061 | 0.235 |
|  | 90 days |  |  |  | 1.000 | 0.958 | 0.876 |
|  | 120 days |  |  |  | 1.000 | 0.895 | 0.861 |
|  | 180 days |  |  |  | 1.000 | 0.999 | 0.963 |
|  | 275 days |  |  |  | 1.000 | 0.998 | 0.839 |
|  | 365 days |  |  |  | 1.000 | 1.000 | 0.999 |
| Stool consistency ^2^ | Overall | <0.0001 | <0.0001 | 0.322 | 0.175 | 0.010 | <0.0004 |
|  | 30 days |  |  |  | 1.000 | 0.274 | 0.149 |
|  | 90 days |  |  |  | 1.000 | 0.380 | 0.229 |
|  | 120 days |  |  |  | 1.000 | 1.000 | 1.000 |
|  | 180 days |  |  |  | 1.000 | 1.000 | 0.979 |
|  | 275 days |  |  |  | 1.000 | 1.000 | 0.997 |
|  | 365 days |  |  |  | 1.000 | 1.000 | 0.979 |

^1^ Group differences throughout the study period were analyzed using mixed models.

^2^ Group differences throughout the study period were analyzed using repeated measures ordinal logistic regressions, modeling the lower score (harder stools texture) of stool consistency.

**SUPPLEMENTARY TABLE 10** Fussiness and gassiness in the past 24 hours of each visit

| **Visit (Age)** | **Outcome** | **Investigational** | **Control** | **Breastfed** | **Overall Group Difference (p-value)** |
| --- | --- | --- | --- | --- | --- |
|  |  |  |  |  |  |
| 30 days | N | 150 | 150 | 150 |  |
|  | Fussiness |  |  |  | 0.690 |
|  | Not at all | 20 (13.33) | 23 (15.33) | 25 (16.67) |  |
|  | Slightly | 104 (69.33) | 104 (69.33) | 102 (68.00) |  |
|  | Moderately | 26 (17.33) | 23 (15.33) | 23 (15.33) |  |
|  | Very | 0 (0.00) | 0 (0.00) | 0 (0.00) |  |
|  | Extremely | 0 (0.00) | 0 (0.00) | 0 (0.00) |  |
|  | Fussiness compared to usual |  |  |  | 0.953 |
|  | Less than usual | 5 (3.33) | 6 (4.00) | 5 (3.33) |  |
|  | Same as usual | 122 (81.33) | 118 (78.67) | 120 (80.00) |  |
|  | More than usual | 23 (15.33) | 26 (17.33) | 25 (16.67) |  |
|  | Gassiness |  |  |  | 0.882 |
|  | Not at all | 0 (0.00) | 0 (0.00) | 0 (0.00) |  |
|  | Slight amount | 17 (11.33) | 18 (12.00) | 20 (13.33) |  |
|  | Moderate amount | 132 (88.00) | 129 (86.00) | 128 (85.33) |  |
|  | Excessive amount | 1 (0.67) | 3 (2.00) | 2 (1.33) |  |
|  | Gassiness compared to usual |  |  |  | 0.179 |
|  | Less than usual | 5 (3.33) | 5 (3.33) | 3 (2.00) |  |
|  | Same as usual | 131 (87.33) | 132 (88.00) | 144 (96.00) |  |
|  | More than usual | 14 (9.33) | 13 (8.67) | 3 (2.00) |  |
| 90 days | N | 132 | 141 | 130 |  |
|  | Fussiness |  |  |  | 0.622 |
|  | Not at all | 6 (4.55) | 9 (6.38) | 10 (7.69) |  |
|  | Slightly | 109 (82.58) | 114 (80.85) | 105 (80.77) |  |
|  | Moderately | 16 (12.12) | 18 (12.77) | 15 (11.54) |  |
|  | Very | 1 (0.76) | 0 (0.00) | 0 (0.00) |  |
|  | Extremely | 0 (0.00) | 0 (0.00) | 0 (0.00) |  |
|  | Fussiness compared to usual |  |  |  | 0.847 |
|  | Less than usual | 9 (6.82) | 9 (6.38) | 6 (4.62) |  |
|  | Same as usual | 92 (69.70) | 102 (72.34) | 94 (72.31) |  |
|  | More than usual | 31 (23.48) | 30 (21.28) | 30 (23.08) |  |
|  | Gassiness |  |  |  | 0.970 |
|  | Not at all | 0 (0.00) | 0 (0.00) | 0 (0.00) |  |
|  | Slight amount | 75 (56.82) | 78 (55.32) | 74 (56.92) |  |
|  | Moderate amount | 57 (43.18) | 63 (44.68) | 55 (42.31) |  |
|  | Excessive amount | 0 (0.00) | 0 (0.00) | 1 (0.77) |  |
|  | Gassiness compared to usual |  |  |  | 0.723 |
|  | Less than usual | 54 (40.91) | 56 (39.72) | 45 (34.62) |  |
|  | Same as usual | 70 (53.03) | 70 (49.65) | 81 (62.31) |  |
|  | More than usual | 8 (6.06) | 15 (10.64) | 4 (3.08) |  |
| 120 days | N | 120 | 127 | 120 |  |
|  | Fussiness |  |  |  | 0.786 |
|  | Not at all | 23 (19.17) | 23 (18.11) | 20 (16.67) |  |
|  | Slightly | 69 (57.50) | 72 (56.69) | 68 (56.67) |  |
|  | Moderately | 28 (23.33) | 31 (24.41) | 32 (26.67) |  |
|  | Very | 0 (0.00) | 1 (0.79) | 0 (0.00) |  |
|  | Extremely | 0 (0.00) | 0 (0.00) | 0 (0.00) |  |
|  | Fussiness compared to usual |  |  |  | 0.839 |
|  | Less than usual | 19 (15.83) | 19 (14.96) | 16 (13.33) |  |
|  | Same as usual | 67 (55.83) | 67 (52.76) | 69 (57.50) |  |
|  | More than usual | 34 (28.33) | 41 (32.28) | 35 (29.17) |  |
|  | Gassiness |  |  |  | 0.132 |
|  | Not at all | 1 (0.83) | 0 (0.00) | 0 (0.00) |  |
|  | Slight amount | 100 (83.33) | 112 (88.19) | 112 (93.33) |  |
|  | Moderate amount | 19 (15.83) | 15 (11.81) | 8 (6.67) |  |
|  | Excessive amount | 0 (0.00) | 0 (0.00) | 0 (0.00) |  |
|  | Gassiness compared to usual |  |  |  | 0.942 |
|  | Less than usual | 38 (31.67) | 40 (31.500 | 36 (30.00) |  |
|  | Same as usual | 72 (60.00) | 78 (61.42) | 79 (65.83) |  |
|  | More than usual | 10 (8.33) | 9 (7.09) | 5 (4.17) |  |
| 180 days | N | 113 | 118 | 110 |  |
|  | Fussiness |  |  |  | 0.726 |
|  | Not at all | 18 (15.93) | 15 (12.71) | 21 (19.09) |  |
|  | Slightly | 59 (52.21) | 62 (52.54) | 56 (50.91) |  |
|  | Moderately | 30 (26.55) | 37 (31.36) | 27 (24.55) |  |
|  | Very | 6 (5.31) | 4 (3.39) | 5 (4.55) |  |
|  | Extremely | 0 (0.00) | 0 (0.00) | 1 (0.91) |  |
|  | Fussiness compared to usual |  |  |  | 0.403 |
|  | Less than usual | 14 (12.39) | 21 (17.80) | 25 (22.73) |  |
|  | Same as usual | 65 (57.52) | 66 (55.93) | 53 (48.18) |  |
|  | More than usual | 34 (30.09) | 31 (26.27) | 32 (29.08) |  |
|  | Gassiness |  |  |  | 0.103 |
|  | Not at all | 0 (0.00) | 0 (0.00) | 0 (0.00) |  |
|  | Slight amount | 103 (91.15) | 113 (95.76) | 107 (97.27) |  |
|  | Moderate amount | 10 (8.85) | 5 (4.24) | 3 (2.73) |  |
|  | Excessive amount | 0 (0.00) | 0 (0.00) | 0 (0.00) |  |
|  | Gassiness compared to usual |  |  |  | 0.343 |
|  | Less than usual | 38 (33.63) | 44 (37.29) | 40 (36.36) |  |
|  | Same as usual | 65 (57.52) | 68 (57.63) | 69 (62.73) |  |
|  | More than usual | 10 (8.85) | 6 (5.08) | 1 (0.91) |  |
| 275 days | N | 113 | 110 | 108 |  |
|  | Fussiness |  |  |  | 0.899 |
|  | Not at all | 18 (15.93) | 16 (14.55) | 20 (18.52) |  |
|  | Slightly | 58 (51.33) | 56 (50.91) | 51 (47.22) |  |
|  | Moderately | 28 (24.78) | 29 (26.36) | 29 (26.85) |  |
|  | Very | 9 (7.96) | 9 (8.18) | 8 (7.41) |  |
|  | Extremely | 0 (0.00) | 0 (0.00) | 0 (0.00) |  |
|  | Fussiness compared to usual |  |  |  | 0.940 |
|  | Less than usual | 17 (15.04) | 17 (15.45) | 14 (12.96) |  |
|  | Same as usual | 74 (65.49) | 74 (67.27) | 76 (70.37) |  |
|  | More than usual | 22 (19.47) | 19 (17.27) | 18 (16.67) |  |
|  | Gassiness |  |  |  | 0.921 |
|  | Not at all | 0 (0.00) | 0 (0.00) | 0 (0.00) |  |
|  | Slight amount | 107 (94.69) | 104 (94.55) | 101 (93.52) |  |
|  | Moderate amount | 6 (5.31) | 6 (5.45) | 7 (6.48) |  |
|  | Excessive amount | 0 (0.00) | 0 (0.00) | 0 (0.00) |  |
|  | Gassiness compared to usual |  |  |  | 0.882 |
|  | Less than usual | 32 (28.32) | 29 (26.36) | 28 (25.93) |  |
|  | Same as usual | 75 (66.37) | 75 (68.18) | 73 (67.59) |  |
|  | More than usual | 6 (5.31) | 6 (5.45) | 7 (6.48) |  |
| 365 days | N | 113 | 110 | 107 |  |
|  | Fussiness |  |  |  | 0.231 |
|  | Not at all | 12 (10.62) | 7 (6.36) | 11 (10.28) |  |
|  | Slightly | 89 (78.76) | 86 (78.18) | 85 (79.44) |  |
|  | Moderately | 11 (9.73) | 16 (14.55) | 11 (10.28) |  |
|  | Very | 1 (0.88) | 1 (0.91) | 0 (0.00) |  |
|  | Extremely | 0 (0.00) | 0 (0.00) | 0 (0.00) |  |
|  | Fussiness compared to usual |  |  |  | 0.361 |
|  | Less than usual | 1 (0.88) | 0 (0.00) | 0 (0.00) |  |
|  | Same as usual | 104 (92.04) | 102 (92.73) | 104 (97.20) |  |
|  | More than usual | 8 (7.08) | 8 (7.27) | 3 (2.80) |  |
|  | Gassiness |  |  |  | 0.368 |
|  | Not at all | 0 (0.00) | 0 (0.00) | 0 (0.00) |  |
|  | Slight amount | 113 (100.00) | 109 (99.09) | 107 (100.00) |  |
|  | Moderate amount | 0 (0.00) | 1 (0.91) | 0 (0.00) |  |
|  | Excessive amount | 0 (0.00) | 0 (0.00) | 0 (0.00) |  |
|  | Gassiness compared to usual |  |  |  | 0.759 |
|  | Less than usual | 9 (7.96) | 7 (6.36) | 8 (7.48) |  |
|  | Same as usual | 104 (92.05) | 102 (92.73) | 99 (92.52) |  |
|  | More than usual | 0 (0.00) | 1 (0.91) | 0 (0.00) |  |

Data presented are count (%). Group differences were analyzed using Cochran-Mantel-Haenszel row-mean score test.

**SUPPLEMENTARY TABLE 11** Group comparisons of fussiness and gassiness in the past 24 hours throughout the study from 30 to 365 days of age (Repeated measures ordinal logistic regression)

| **Outcome** | **Model Effect (p-value)** | | |
| --- | --- | --- | --- |
|  | **Group** | **Age** | **Group*Age Interaction** |
| Fussiness | 0.598 | 0.0003 | 0.881 |
| Fussiness compared to usual | 0.892 | <0.0001 | 0.916 |
| Gassiness | 0.246 | <0.0001 | 0.545 |
| Gassiness compared to usual | 0.740 | <0.0001 | 0.802 |

Group differences throughout the study period from 30 to 365 days of age were analyzed using repeated measures ordinal logistic regression models, modeling the lower level of fussiness or gassiness.

**SUPPLEMENTARY TABLE 12** Study formula intake in the past 24 hours of each visit

| **Visit (Age)** | **Boys** | | | | **Girls** | | | |
| --- | --- | --- | --- | --- | --- | --- | --- | --- |
|  | **Investigational (mL/d)** | **Control (mL/d)** | ^1^ **Breast milk intake (g/d)**  **[*M* (P25, P75)]** | **Group Difference (p-value)**  **Investigational vs. Control** | **Investigational (mL/d)** | **Control**  **(mL/d)** | ^1^ **Breast milk intake (g/d)**  **[*M* (P25, P75)]** | **Group Difference (p-value)**  **Investigational vs. Control** |
| 90 days | 806±103 | 794±110 | 850 (704, 1090) | 0.529 | 755±94 | 756±93 | 827 (690, 986) | 0.946 |
| 120 days | 857±90 | 854±86 | 961 (750, 1235) | 0.851 | 820±70 | 809±70 | 903 (726, 975) | 0.394 |
| 150 days |  |  | 986 (790, 1050) |  |  |  | 702 (620, 1910) |  |
| 180 days | 891±79 | 878±88 |  | 0.403 | 868±73 | 851±67 |  | 0.190 |
| 275 days | 945±72 | 929±74 |  | 0.269 | 927±62 | 922±59 |  | 0.691 |
| 365 days | 786±182 | 774±132 |  | 0.682 | 761±136 | 747±197 |  | 0.662 |

Data presented are mean ± standard deviation. Group differences were analyzed using analysis of variance.

^1^ The data on breast milk intake referred to a survey conducted by the Chinese Center for Disease Control and Prevention on Breast milk intake of Chinese infants aged 0-5 months in 2019-2021(33).

**SUPPLEMENTARY TABLE 13** Number of subjects with adverse events during the study period

| **Adverse Event** | **Investigational (*n*=150)** | **Control (*n*=150)** | **Breastfed (*n*=150)** | **Group Difference p-value** |
| --- | --- | --- | --- | --- |
| **Blood and lymphatic system disorders** | | | |  |
| Anemia | 2 (1.33) | 4 (2.67) | 10 (6.67) | 0.059 |
| Fever | 39 (26.00) | 38 (25.33) | 33 (22.00) | 0.701 |
| **Cardiovascular system disorders** | | | |  |
| Heart murmur | 0 (0.00) | 2 (1.33) | 1 (0.67) | 0.776 |
| **Ear and labyrinth disorders** | | | |  |
| Otitis media | 3 (2.00) | 4 (2.67) | 1 (0.67) | 0.546 |
| **Gastrointestinal disorders** | | | |  |
| Emesis / Vomiting | 22 (14.67) | 25 (16.67) | 13 (8.67) | 0.100 |
| Gas | 21 (14.00) | 24 (16.00) | 17 (11.33) | 0.525 |
| Diarrhea | 21 (14.00) | 22 (14.67) | 10 (6.67) | 0.056 |
| Constipation | 10 (6.67) | 13 (8.67) | 8 (5.33) | 0.553 |
| Acute gastroenteritis | 1 (0.67) | 0 (0.00) | 0 (0.00) | 1.000 |
| Anal fissure | 0 (0.00) | 0 (0.00) | 1 (0.67) | 1.000 |
| Umbilical hernia | 3 (2.00) | 4 (2.67) | 4 (2.67) | 1.000 |
| **Skeletal muscle system disorders** | | | |  |
| Torticollis | 6 (4.00) | 3 (2.00) | 1 (0.67) | 0.172 |
| **Respiratory, thoracic and mediastinal disorders** | | | |  |
| URI | 44 (29.33) | 47 (31.33) | 41 (27.33) | 0.761 |
| Cough with no other related symptoms | 5 (3.33) | 4 (2.67) | 4 (2.67) | 1.000 |
| Bronchitis | 2 (1.33) | 0 (0.00) | 2 (1.33) | 0.553 |
| Pneumonia | 4 (2.67) | 4 (2.67) | 5 (3.33) | 1.000 |
| **Skin and subcutaneous tissue disorder** | | | |  |
| Diaper rash | 41 (27.33) | 37 (24.67) | 39 (26.00) | 0.884 |
| Dry skin | 6 (4.00) | 8 (5.33) | 7 (4.67) | 0.960 |
| Eczema / Atopic dermatitis | 25 (16.67) | 33 (22.00) | 35 (23.33) | 0.317 |
| Seborrhea / Cradle cap | 32 (21.33) | 33 (22.00) | 28 (18.67) | 0.758 |
| **Renal and urinary disorders** | | | |  |
| Hydrocele | 4 (2.67) | 0 (0.00) | 1 (0.67) | 0.133 |
| **Other** | | | |  |
| Limited hip motion | 8 (5.33) | 9 (6.00) | 10 (6.67) | 0.968 |
| Asymmetric dermatoglyph | 7 (4.67) | 12 (8.00) | 7 (4.67) | 0.406 |
| HFMD | 1 (0.67) | 0 (0.00) | 0 (0.00) | 1.000 |

Data presented are frequency (%). Group differences were analyzed using Fisher’s exact test.


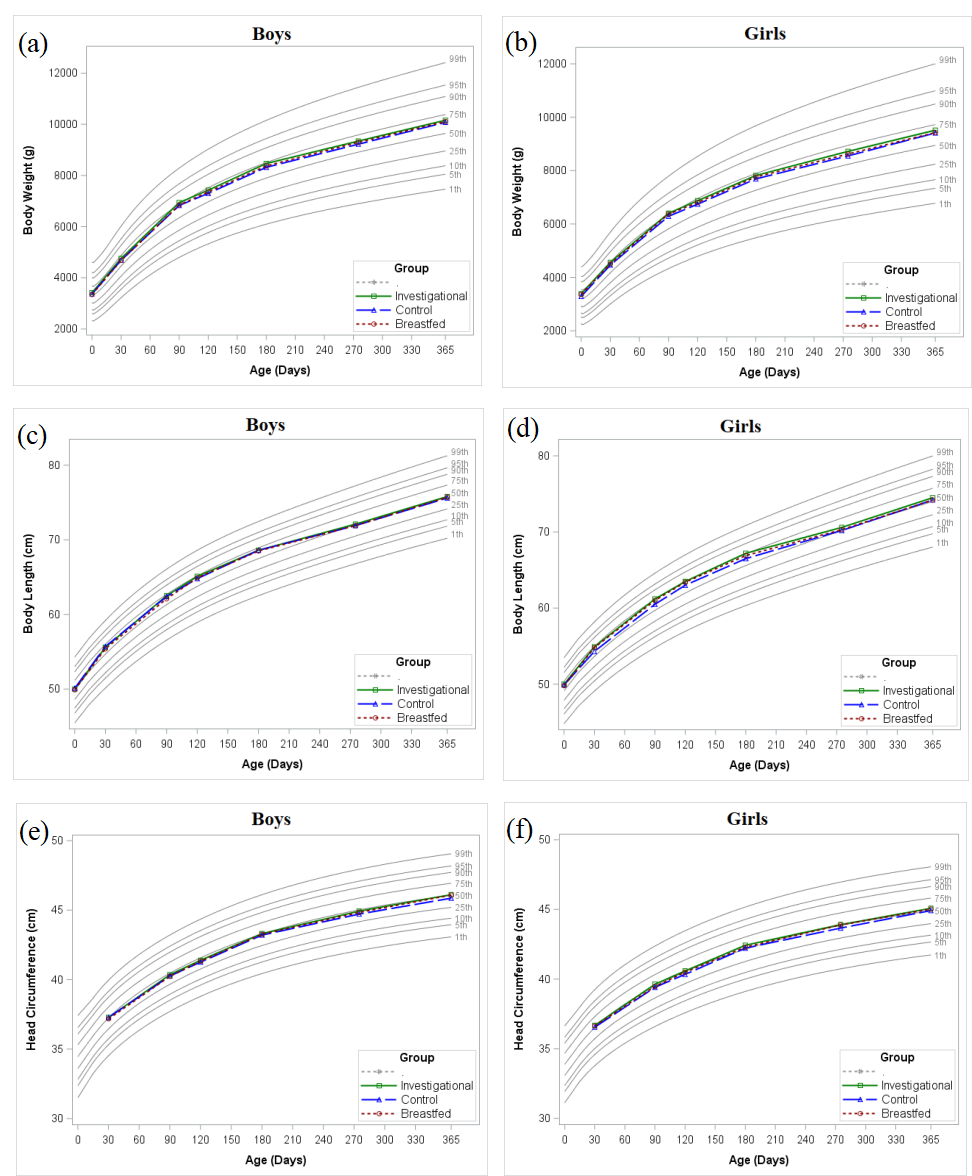


**SUPPLEMENTARY FIGURE 1** Mean achieved weight, length and head circumference for participants through 365 days of age. Investigational, green square; Control, blue triangle; Breastfed, red circles. (a) Mean achieved weight for boy participants with world health organization reference percentiles (1st to 99th) through 365 days of age. (b) Mean achieved weight for girl participants with world health organization reference percentiles (1st to 99th) through 365 days of age. (c) Mean achieved length for boy participants with world health organization reference percentiles (1st to 99th) through 365 days of age. (d) Mean achieved length for girl participants with world health organization reference percentiles (1st to 99th) through 365 days of age. (e) Mean achieved head circumference for boy participants with world health organization reference percentiles (1st to 99th) through 365 days of age. (f) Mean achieved head circumference for girl participants with world health organization reference percentiles (1st to 99th) through 365 days of age.
